# Understanding school food systems to support the development and implementation of food based policies and interventions

**DOI:** 10.1186/s12966-023-01432-2

**Published:** 2023-03-13

**Authors:** Maria Bryant, Wendy Burton, Niamh O’Kane, Jayne V. Woodside, Sara Ahern, Phillip Garnett, Suzanne Spence, Amir Sharif, Harry Rutter, Tim Baker, Charlotte E. L. Evans

**Affiliations:** 1 Department of Health Sciences, University of York, York, YO150DD UK; 2 Hull York Medical School, University of York, York, YO150DD UK; 3 Centre for Public Health, Queen’s University Belfast, Belfast, BT12 6BJ UK; 4 Bradford Institute of Health Research, Bradford Royal Infirmary, Bradford, BD9 6RJ UK; 5 School for Business and Society, University of York, York, YO10 5DD UK; 6 Human Nutrition Exercise Research Centre, Population Health Sciences Institute, Newcastle University, Newcastle upon Tyne, NE2 4HH UK; 7 Faculty of Management, Law and Social Sciences, University of Bradford, Bradford, BD7, 1DP UK; 8 Department of Social and Policy Sciences, University of Bath, Bath, BA2 7AY UK; 9 Charlton Manor Primary School, Indus Road, Charlton, London, SE7 7EF UK; 10 School of Food Science and Nutrition, University of Leeds, Leeds, LS2 9JT UK

**Keywords:** School, Food, System, Map, Stakeholder, Environment, Network, Children, Diet quality

## Abstract

**Background:**

Schools provide opportunities to improve the quality of children's diet, whilst reducing inequalities in childhood diet and health. Evidence supports whole school approaches, including consistency in food quality, eating culture and food education. However, such approaches are often poorly implemented due to the highly complex environments in which schools operate. We aimed to develop a school food systems map using a systems thinking approach to help identify the key factors influencing primary school children’s dietary choice.

**Methods:**

Eight workshops were conducted with 80 children (from schools from varying locations (region of England/UK; urban/rural), deprivation levels and prioritisation of school food policies)) and 11 workshops were held with 82 adult stakeholders across the UK (principals, teachers, caterers, school governors, parents, and local and voluntary sector organisations) to identify factors that influence food choice in children across a school day and their inter-relationships. Initial exploratory workshops started with a ‘blank canvas’ using a group model building approach. Later workshops consolidated findings and supported a wider discussion of factors, relationships and influences within the systems map. Strengths of the relationship between factors/nodes were agreed by stakeholders and individually depicted on the map. We facilitated an additional eight interactive, in-person workshops with children to map their activities across a whole school day to enable the production of a journey map which was shared with stakeholders in workshops to facilitate discussion.

**Results:**

The final ‘CONNECTS-Food’ systems map included 202 factors that were grouped into 27 nodes. Thematic analysis identified four key themes: leadership and curriculum; child food preference; home environment; and school food environment. Network analysis highlighted key factors that influence child diet across a school day, which were largely in keeping with the thematic analysis; including: 'available funds/resources', 'awareness of initiatives and resources', 'child food preference and intake', 'eligibility of free school meals', 'family circumstances and eating behaviours', 'peer/social norms', 'priorities of head teachers and senior leaders'.

**Conclusions:**

Our systems map demonstrates the need to consider factors external to schools and their food environments. The map supports the identification of potential actions, interventions and policies to facilitate a systems-wide positive impact on children’s diets.

**Supplementary Information:**

The online version contains supplementary material available at 10.1186/s12966-023-01432-2.

## Background

Around 30% of foods and drinks consumed by children are consumed during the school day (Nathan et al*.* 2019); providing an opportunity for schools to improve dietary quality and reduce inequalities in obesity and health. This is important, as dietary intake consistently fails to meet government recommendations (e.g. [1]). Children aged 4–11 years consume above recommended intakes of free sugars and saturated fat and inadequate amounts of fibre, vitamin D and fruits and vegetables. Furthermore, substantial dietary inequalities exist in the UK, particularly affecting those living in the most deprived households, who are twice as likely to have obesity [2] and less likely to achieve dietary guidelines [3, 4]).

The WHO’s Health Promoting Schools framework advocates a whole school approach to promoting health [5]. In England, current government initiatives include mandated food based school food standards [6] and similar standards are mandated in other nations (e.g. [7, 8]. Previous research that has explored the impact of such legislated food and nutrient-based standards has highlighted the potential positive impact on primary school children's dietary intake [9, 10]. However, non-mandated recommendations, including ‘whole school approaches to food’ have an under-realised potential to improve children’s eating habits within and outside of school [11], with evidence indicating poor implementation (failure to engage parents and no consideration of sustainability) and evaluation (limited data on long-term effects, system adaptation or contextual factors) [10, 12]. This is an area gaining increased interest, including within the UKs Levelling Up White paper. Notwithstanding the political uncertainties, this is a policy paper which recommends that schools not only improve their whole school approaches to food, but provide a statement of this on their websites [13]. These ‘whole school approaches’ advocate a systems approach to the provision and education surrounding food, including the promotion of a consistent food culture in schools (how food is provided and the ethos around celebration foods [14], food policy (such as regulations on packed lunches) and education (healthy food practices within the curriculum).

Application of systems thinking is well established in many policy areas, but the use of systems approaches to improve population health including food environments is relatively new [15]. Key aspects of complex systems thinking are identification of connections and strengths of relationships between different parts of the system and the need to see the system from many different points of view [15]. Although a systems approach is considered useful for designing policies that take account of the complexities involved, it does lead to large amounts of inherent unpredictability [16]. Nevertheless, some countries, including the UK, encourage systems approaches in public health policy due to their potential benefits [4].

There are many competing priorities and demands within schools and it is not clear how whole school approaches to food fit within a broader context of school based health promotion. There is further complexity found within the wider educational system, in which decision making is often linked to the delegation of funding and responsibility to schools, and the increase in numbers of independent academies, and changes in the wider food system beyond the school environment. Whilst a number of initiatives for enhancing food environments within schools exist, uptake has been low [17], limiting the potential for demonstrable impacts on diet and health. Thus, schools would benefit from active support to deliver effective policies/guidance which support whole school approaches to food.

Systems-led work in schools in Canada suggests that there are three key factors that may influence the ability of school food interventions to have an impact, including the actions of key staff (“Actors and Elements”), the implementation of different school food policies (“System Regulation and Interconnections”) and priorities of stakeholders (“Purpose and Values”) [18]. This is likely to resonate with school food systems in the UK, where existing implementation evaluation of the School Food Plan suggests that the skill and will of head teachers is a strong predictor of success [19]. Further evidence suggests that government incentives and commitment from multiple stakeholders is required to achieve a higher uptake of guidance of school plans [20]. Case studies highlighted by ‘what works well’ within the School Food Plan offer some additional insight to guide optimisation of the whole schools approaches, but there has been a lack of evaluation of potential impacts [20]. Development of interventions to optimise school food provision and consumption requires an understanding of local and wider influences on the school food system and potential levers to shape them [21]. However, the paucity of research on systems approaches to school food has potentially hindered the development, and evaluation, of whole school approach interventions.

This study attempted to fill the gap in evidence within school food system through the development of a primary school food systems map. This was intended to highlight key factors influencing children’s food choice across a school day in the UK, in order to support the design of school food interventions or policies, including those that support the implementation of whole school approaches to food. This adopted a co-design approach alongside key stakeholders in order to identify complex and non-linear pathways through which decision making occurs in schools, including key organisational components and/or political pathways for successful implementation of whole school approaches to food. Central to this was the decision making at the level of the child. Hence, we were interested in mapping the system to allow for the identification of opportunities within the system that could influence child food choice via whole school approaches to food.

## Methods

### Aim

To build a systems map of influences on school children’s food choice throughout the school day, performing network analysis to describe relationships, complexity, interactions and potential adaptations through our mapping activities—generating theories and assumptions required to support future intervention development in this setting.

### Study design

We used a group model building approach [22, 23] to develop our systems map. This is a participatory approach in which a group of stakeholders with differing perspectives are brought together to build a shared understanding of a complex system. In addition to stakeholder workshops, eight separate workshops were also planned with primary school children to provide an understanding of key experiences throughout a child's school day with the potential to influence food choice either directly (i.e. via the offer of food) or indirectly (e.g. exposure to foods/food marketing). These ‘journey mapping’ workshops were interspersed with workshops with school stakeholders to support discussions. Earlier, ‘phase 1’ workshops were exploratory in nature and elicited factors that influenced the child’s journey across the school day and their inter-relationships, resulting in the development of an initial systems map. Later, confirmatory, ‘phase 2’ workshops consolidated and refined the findings, enabling the development of the final systems map.

Ethical approval for the study was received from the University of York Department of Health Sciences’ Research Governance Committee (ref HSRGC/20210/428/A) and we used the Consolidated Criteria for Reporting Qualitative Research (COREQ) checklist to guide our approach and reporting (Tong, 2007).

### Site selection and participant recruitment

Research was undertaken in four regions of the UK (Leeds, Bradford, Newcastle, Belfast) led by a principal investigator (PI) in each site (SA/CE/JW/SS). However, as the majority of adult stakeholder workshops were delivered remotely, relevant stakeholders outside of these areas were also invited to take part (e.g. national and local organisations with a key role in school food). All childhood workshops were planned to be delivered in person. The following eligibility criteria were applied:

#### Child workshop: Inclusion criteria

- Children from any year group were eligible. We engaged with existing school councils / food ambassadors / global champions to enable a breadth of engagement across the school

#### Stakeholder workshop: Inclusion criteria

- Public and academy schools across a range of differing demographics with single and multiple form entry
- School stakeholders to include: head teachers, teaching staff, catering staff, school governors, and parents
- External catering stakeholders to include: representatives from catering and/or procurement services, and food supply chain agents (producers, distributors)
- External businesses (as appropriate) including local businesses

#### Child workshop: Exclusion criteria

- Children whose families did not provide consent for them to take part

#### Stakeholder workshop: Exclusion criteria

- Private and specialist schools

Stakeholders were invited to take part via direct communication with head teachers from schools within each area. We also promoted the study using poster/leaflet and through direct invitations from those that had already agreed to take part (snowballing method [24]. Social media (Twitter) and existing networks (e.g. GENIUS school food network (https://geniusschoolfoodnetwork.com/)) were then used to invite stakeholders from outside of the immediate schools that had an interest in school food. All workshop participants were required to provide written informed consent prior to taking part. There was no limit placed on the number of people recruited to each workshop, although we anticipated 10–12 people per workshop. Further, stakeholders across the four regions were able to attend any workshop (i.e. they were not restricted to workshops that were organised within their region). Children were invited to take part by school teachers and written informed consent was obtained from parents / guardians prior to workshops. All child workshops were delivered during a school day, within two schools in each region.

### Sampling

A sampling framework was applied in attempt to ensure a diversity of the eight planned schools according to area level deprivation (at least four schools situated within the highest quintiles of deprivation from the index of multiple deprivation), with a range in geography (urban and rural locations) and the level of school engagement with school food initiative (defined as either having an existing school food strategy or not) (Additional file 3).

### Child journey mapping workshops

Journey mapping is typically used in healthcare to map the patient journey and inform service improvement [25, 26]. For this study, a card based activity was performed with children by two members of the research team to build a picture of a typical day in the life of a primary school child by asking children to talk about key timepoints throughout the previous day. This information was used Although dietary choices were the main focus of the research, facilitators did not make them the main focus of the discussion in order to remove potential anxiety around describing personal food habits and to allow for the identification of a broader range of events during a day which have the potential to influence food choice (e.g. exposure to food marketing during the journey to school, attending sports clubs etc.). In each workshop, children were asked to pick a card that denoted a particular time in the day (e.g. waking up and getting ready for school, lunch time, during lessons etc.) and asked to describe what they did the day before at this time point. Once the child had responded to their card, the question was opened out to all children. During discussions, responses were mapped onto a whiteboard at the appropriate time in the day by the support facilitator.

### Exploratory systems mapping workshops with stakeholders

We originally planned to deliver eight × 90 min workshops in total, four exploratory and four confirmatory, although this was updated to seven and three workshops respectively with agreement within the team, as the exploratory workshops continued to elicit new information beyond the planned four sessions. These initial exploratory workshops used a ‘blank canvas’ approach to identify factors that influence food choice by children during the school day using a group model building approach [22].

At the start of the exploratory workshops, participants were introduced to the systems mapping concept, the aim of the workshop and informed that they would be helping to develop a map of the school food system. Journey maps produced from the child workshops were presented to prompt discussion. In the first half of the workshop, participants were divided into small groups (ranging from 2 – 6 people) and given two minutes to think independently of 1–3 factors that influenced child food choice/dietary habits throughout the school day. Participants then shared these factors with the rest of their small group in turn and these were recorded by a study note taker on an interactive whiteboard (Google Jamboard) using the sticky note function. Discussions around the factors were then held, with participants asked to expand on how they felt that the factors influenced child food choice dietary habits. The wider group was then reconvened in a plenary discussion where participants were asked to comment on each other's choices. In the second half of the exploratory workshops, participants, in small groups again, were asked to consider if any of the factors were related to each other and if so, how (including the direction of relationship). Discussions during this activity were captured on the same interactive whiteboard by drawing directional arrows between the sticky notes. Additional factors revealed during this discussion were also added to the jamboard.

### Development of initial systems map

Following all exploratory workshops, interactive whiteboards completed during the workshops were reviewed and an overall list of factors that stakeholders believed influence child dietary choice and food habits was compiled. Recordings of the workshops were reviewed to ensure no factors had been overlooked. These were then thematically grouped by two members of the research team (WB & NOK), with each group being assigned a theme name and descriptive summary. Overarching themes were also identified and agreed initially by WB & NOK and then by the rest of the team. Theme names were entered onto a matrix using Microsoft Excel. One member of the research team (WB) again reviewed data collected via interactive whiteboards and discussions during the workshops to identify themes that were connected to one another, and the direction of the relationships. Linked themes were represented visually on the matrix which was reviewed by a second member of the research team (NOK). The STICKE map builder application (Deakin University 2019 STICKE [software] (build 640—Oct 2019) [Accessed 2021]) was used to develop the initial systems map. Each theme was represented by a node on the map and each connection depicted with a directional arrow. Overarching themes were represented as a colour coded domain to assist with interpreting the map. Once the initial map was produced, it was reviewed by the rest of the research team to “sense check” and ensure it was an accurate reflection of workshop discussions.

### Confirmatory workshops

Confirmatory workshops led by a session chair (with two session facilitators) were carried out with stakeholders to consolidate findings and support a wider discussion of factors, relationships and influences within the emergent systems map. Participants were divided into small groups and asked to look in detail at the initial systems map developed from earlier workshops. We applied a focused approach, where participants were asked to review specific areas of the map in turn, followed by the map as a whole, and discuss whether they agreed with the factors/nodes depicted, proposed relationships, and the direction of the relationships. In addition, participants were asked to consider the strength of the relationships between factors (high, medium or low) and whether any nodes or potential relationships were missing.

### Final systems map development

Following completion of the confirmatory workshops, recordings of the workshops were reviewed to ensure all new data captured during discussions were included within the existing nodes and domains on the initial version of the map. This led to the initial thematic groupings to be refined resulting in a revised relationship matrix and a revised version of the map. During the review of workshop recordings, discussions around relationship strengths were also drawn out, allowing relationships to be coded as high, medium, or low in strength on the theme/relationship matrix. New connections identified during workshop discussions were also incorporated. Following refinement of the map, it was again reviewed by the wider research team to ensure it coherently depicted concepts and relationships identified during workshops and by a selection of the workshop stakeholders (Headteacher *n* = 4; local authority food leads *n* = 3; catering representatives *n* = 3; representatives of school food organisations *n* = 2; dietician *n* = 1) (who were later involved in using the map to co-design an implementation intervention to support whole school approaches to food). Following this, the final version of the’CONNECTS-Food’ systems map was produced.

### Network analysis

A network analysis was performed to understand and describe relationships, complexity and interactions with the school food systems map developed via the workshops [27]. The theme/relationship matrix developed during development of the systems map was exported as a CSV file and imported into R statistical software as an adjacency matrix. The matrix included edge weights 1—4, with a weight of 1 indicating that there is a relationship between the nodes but the weight is unknown. Weights 2—4 presented low, medium, and high strength relationships respectively.

Within R, iGraph was used for the network analysis by generating a directed graph from the adjacency matrix. We calculated the network density, reciprocity, centrality, and the mean path length. We also calculated the betweenness centrality, in and out degree, and the degree for each of the nodes. Betweenness centrality measures the number of paths between any two nodes that go through the other nodes. Therefore, betweenness centrality in this case provides an indication of how important a node is for connecting together the different influences on children’s food choices. In degree is a count of the number of relationships into a given node from other nodes. Out degree is the number of relationships out from a given node into the other nodes.

We also clustered the network using the cluster edge betweenness algorithm in iGraph to split the network into communities [28]. This clustering method is useful as it can take into account both the direction and weights of the edges. The clusters therefore represent factors that more closely influence each other in the network.

In addition to the clustering of the network we calculated the 'in' coreness of the directed graph by doing a core-periphery analysis using the k-core decomposition algorithm provided by iGraph graph.coreness [27, 29]. This allowed us to determine the core of the network by the connections into the different factors. In this case there are three layers to the core-periphery analysis: (1) A central core of factors that have many relationships into each other; (2) A middle layer that is not as highly connected, but has connections going into the central core; (3) An outer layer of factors that can be considered as more peripheral.

## Results

### Child workshops

In-person workshops were conducted with 80 children from eight schools across four locations with representation according to rurality: (4 = rural, 4 = urban), deprivation (5 = school's IMD < 8, 3 school’s IMD ≥ 8 (or NI equivalent)) and prioritisation of school food policies (*n* = 2 schools yes, *n* = 6 schools unknown) between May–July 2021. A journey map was created after the first workshop and built upon in subsequent workshops so that the information shared by children was available to present in all stakeholder workshops (which were interspersed with child workshops). Children shared their experiences across a whole range of activities within a school day. This enabled us to get a sense of the factors that had a role in child food choice from the perspective of the child. Details of this work will be published separately; but in summary, key discussion points centred around the impact of rurality (particularly related to factors that children experienced on their journey to school), eating behaviours at home, school eating environment and preferences for school or packed lunch. The full range of activities reported by children was provided to stakeholders to enable them to consider multiple factors that might influence child food choice across the day.

### Adult stakeholder workshops

Remote workshops were conducted with 81 adult stakeholders within the same period across 11 sessions. This included an ‘out of hours’ workshops to accommodate those who could not attend workshops during working hours and a workshop attended only by teaching assistants from one of the participating schools who were also not able to attend any of the planned sessions. Participants included those who were based within the four regions, in addition to national school food stakeholders and included representatives from teaching staff, caterers/food producers, lunch time staff, headteachers, governors and parents (Table 1).

**Table 1:** Workshop participants

Group concept mapping workshops—participant type	*N* = 81
Teaching staff	*N* = 23 (Age range 25–64; 22 female, 1 male; 18 white British, 4 Asian/British Asian, 1 undeclared ethnicity)
Catering/lunch staff	*N* = 17 (Age range 35–64; 17 female; all white British/Irish)
Parent	*N* = 11 (Age range 35–64; 9 female, 2 male; all white British/Irish)
School governor	*N* = 7 (Age range 35–65 + ; 7 female; all white British)
Headteacher	*N* = 3 (Age range 50–64; 2 female, 1 male; all white British)
Food producer/distributor	*N* = 1 (Age range 35–49; female, white British/Irish)
Other (including representatives from local authorities, civil servants, school food organisations, nutritionists/dieticians)	*N* = 19 (Age range 18–65 + ; 14 female, 4 male; all white British/Irish)

### Systems map

**Fig. 1:**
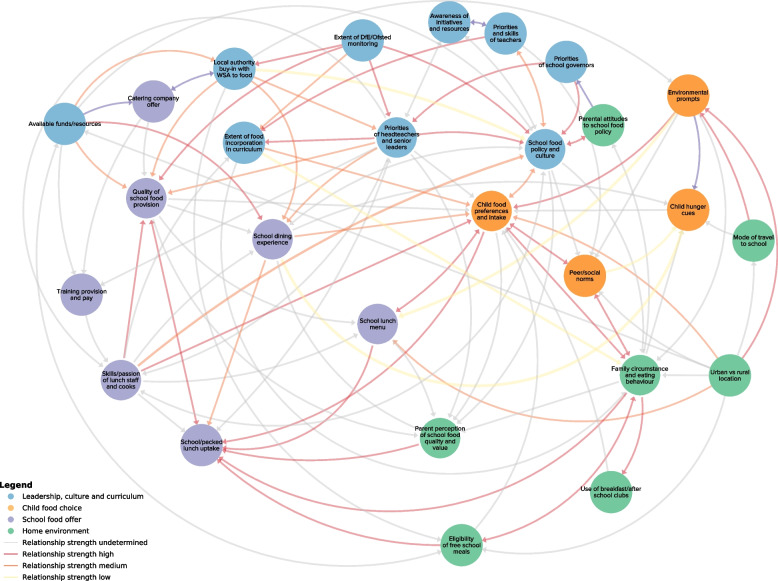
CONNECTS-Food School Food Systems Map

Our systems mapping workshops identified 202 factors which were grouped into 27 thematic nodes. These are represented in our final CONNECTS-Food school food systems map (Fig. 1).

#### Thematic analysis

Thematic analysis identified four key themes: (1) leadership and curriculum; (2) child food preference; (3) home environment; and (4) school food environment (additional file 2). The leadership and curriculum domain comprises nine factors, (e.g. priorities of headteachers and senior leaders, school food policy and culture and the extent of Department for Education (DfE) and Office for Standards in Education, Children's Services and Skills (Ofsted) monitoring). Child food preference comprises four factors which include child food preference and intake, environmental prompts and peer/social norms. The home environment comprises seven factors, which include family circumstances and eating behaviours and parental attitudes to school food policies, and finally, the school food environment domain comprises seven factors, including the quality of school food provision, the school dining experience and the skills and passion of lunchtime staff and cooks. Although it was attempted during confirmatory workshops to ascertain strengths of the relationships between all factors, in practice, this was not possible, as some relationship strengths were difficult to quantify, resulting in an incomplete data set. Where relationships strengths were estimated by stakeholders, these data were used in the network analysis for clustering purposes.

#### Network analysis

**Fig. 2:**
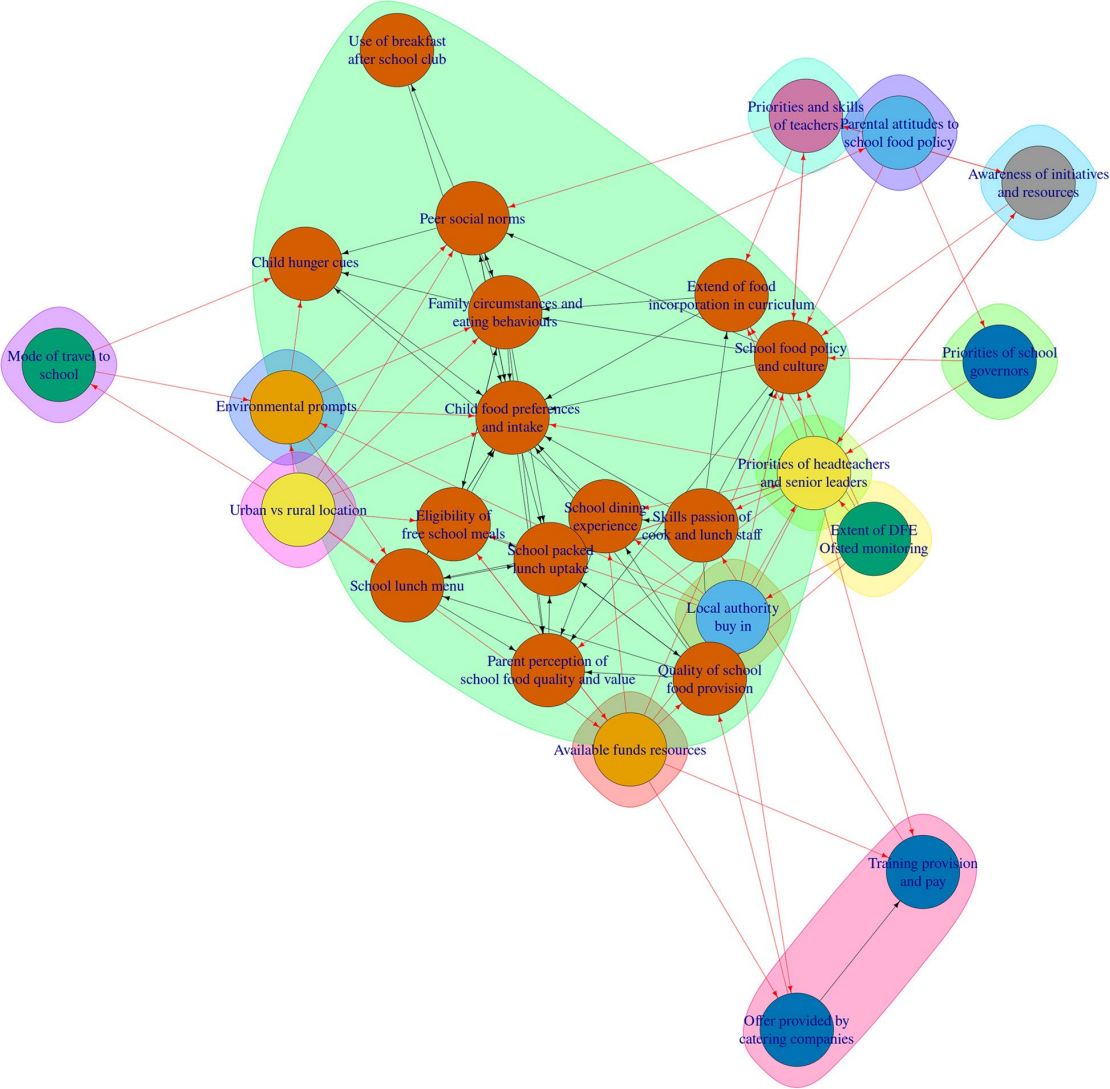
CONNECTS-Food Cluster Map

The results of the network analysis are presented in Table 2. Results show that there are a many factors that rank highly on most network measures, including: 'available funds resources', 'awareness of initiatives and resources', 'child food preference and intake', 'eligibility of free school meals', 'family circumstances and eating behaviours', 'peer social norms', 'priorities of headteachers and senior leaders', 'school food policy and culture', 'school packed lunch uptake', and 'skills passion of cook and lunch staff'. All of these factors scored highly on betweenness and degree measures; suggesting these factors are viewed as significant to children's food choice. Seven out of these 10 highly ranked factors also cluster together into cluster six, along with two other factors that score highly, 'child hunger clues' and 'quality of school food provision' (Fig. 2). Broadly, factors within cluster six describe the potential for children to access healthy food, as they are often either about preferences, availability, or resources (either directly in the form of money, or indirectly in the form of the allocation/prioritisation of resources).

**Table 2:** Results of network analysis

Node Name	Betweenness	Degree	In Degree	Out Degree	Clusters	Coreness
**Available funds resources**	**90.17**	**9**	**2**	**7**	**1**	**1**
**Awareness of initiatives and resources**	**72.67**	**5**	**2**	**3**	**8**	**2**
**Child food preferences and intake**	**107.37**	**18**	**14**	**4**	**6**	**2**
Child hunger cues	29.00	6	5	1	6	2
**Eligibility of free school meals**	**96.90**	**8**	**4**	**4**	**6**	**1**
Environmental prompts	9.53	8	3	5	9	1
Extent of food incorporation in curriculum	24.33	6	4	2	6	2
Extent of DFE Ofsted monitoring	0.00	5	0	5	3	0
**Family circumstances and eating behaviours**	**218.90**	**14**	**6**	**8**	**6**	**2**
Local authority buy in	26.33	9	2	7	2	1
Mode of travel to school	0.50	3	1	2	11	0
Offer provided by catering companies	11.20	4	2	2	13	1
Parent perception of school food quality and value	0.00	8	7	1	6	2
Parental attitudes to school food policy	33.00	3	1	2	10	1
**Peer social norms**	**69.83**	**9**	**6**	**3**	**6**	**2**
Priorities and skills of teachers	14.83	6	2	4	7	2
**Priorities of headteachers and senior leaders**	**141.50**	**15**	**5**	**10**	**4**	**2**
Priorities of school governors	9.50	3	1	2	5	1
Quality of school food provision	37.20	12	7	5	6	2
School dining experience	11.33	9	5	4	6	2
**School food policy and culture**	**90.70**	**14**	**9**	**5**	**6**	**2**
School lunch menu	1.00	8	5	3	6	2
**School packed lunch uptake**	**130.00**	**11**	**9**	**2**	**6**	**2**
**Skills passion of cook and lunch staff**	**72.17**	**9**	**2**	**7**	**6**	**1**
Training provision and pay	25.83	4	3	1	13	1
Urban vs rural location	0.00	8	0	8	12	0
Use of breakfast after school club	0.00	2	1	1	6	1

Bolded rows indicate factors which were ranked highly on most measures. To note, absolute values are based on the total number of factors; thus, they do not represent any given cut-offs

**Fig. 3:**
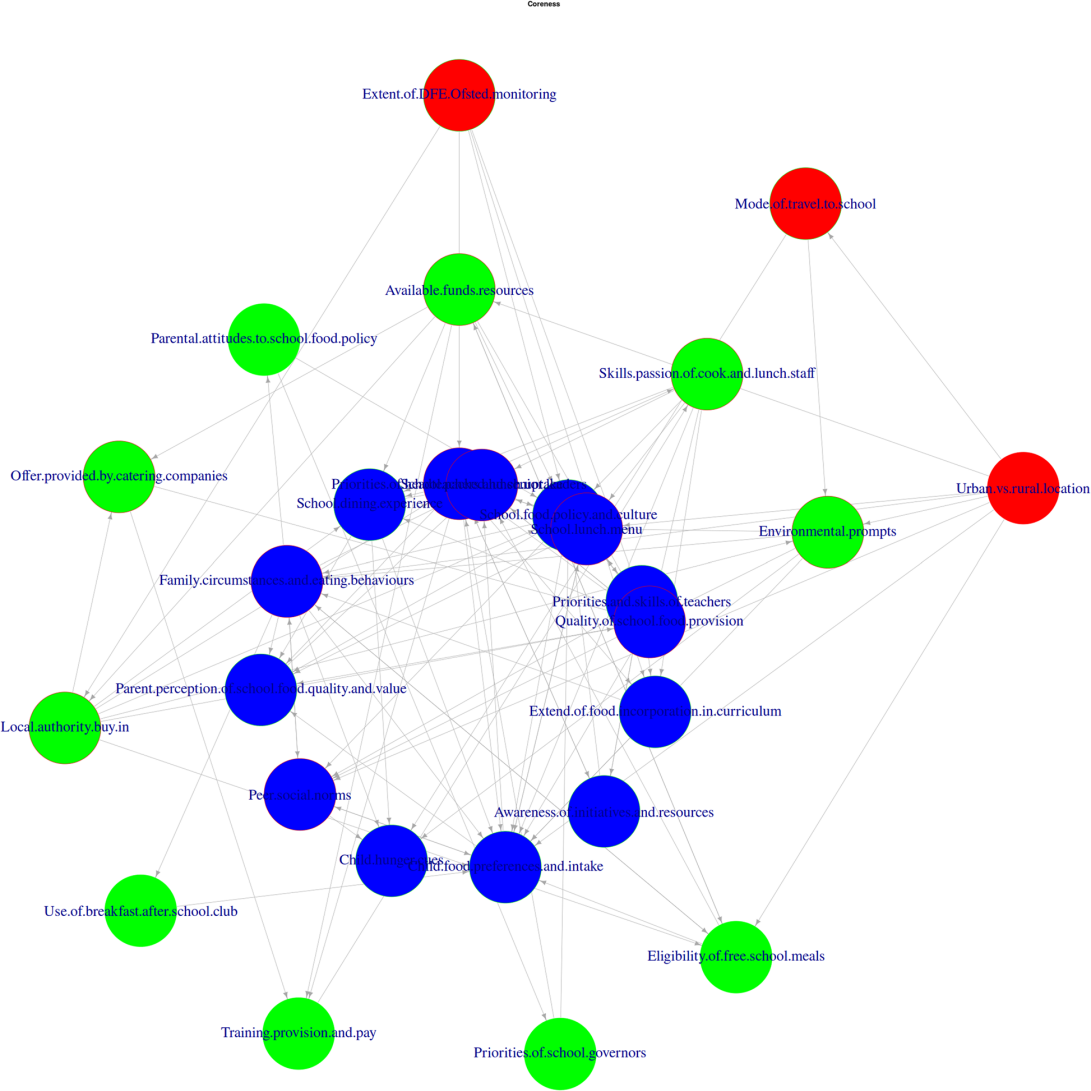
CONNECTS-Food Coreness Map

The core-periphery analysis conducted on the directed network, groups factors into the network core taking the direction of the relationship into account. This produced a core of 14 factors (coreness ‘2’ in Table 2) at the centre of the network shown in Fig. 3, (10 of which are also present in cluster 6). These 14 central core factors also relate to the ability for children to access good food, and additionally include awareness factors such as ‘extent of food incorporation in curriculum’ and ‘parent perception of school food quality and value’. A further 10 factors group around, or influence, this central core (coreness ‘1’ in Table 2), which are broadly more external, such as ‘priorities of school governors’, training provision and pay’, and ‘environmental prompts’ and ‘resource availability’.

## Discussion

Our ‘CONNECTS-Food’ systems map provides an in-depth understanding of the primary school food system through the eyes of a range of key stakeholders. By highlighting factors with potential influence on children’s diets, the map also supports the identification of leverage points which could be used to influence the system it depicts in ways that could promote healthier diets. This includes consideration of both school level and external factors that influence the diets of children. Such information will support the design of future interventions to improve the school food environment. For example, the map has already been used to co-design resources for schools to implement whole school approaches to food in the UK (www.connects-food.com) in combination with the Action Scales Model [30]. In this, the map was used by the co-design team to identify key factors that are likely to influence child diet in schools (e.g. prioritisation of food within senior leaders). Once identified, the map was used to estimate what other factors might need to change to influence this factor (i.e. via relationships within the map). In the case of leadership prioritisation, the map suggests that external monitoring and priorities of school governors are likely influencers. Further, the map tells us that an intervention that is able to impact on school food prioritisation by leadership is likely to have a direct impact on factors such as the quality of food, the school dining experience, training provision and pay, and child food preferences (Additional file 1).

Our network analysis identified 10 key factors that were consistent across all metrics, including those that significantly link to other factors (betweenness), and those that were central to children's food choice (degree). Many of these clustered together and were related to food preferences, availability, or resources (either directly in the form of money, or indirectly in the form of the allocation/prioritisation of resources). This was consistent with our thematic analysis, in which key themes linked to leadership (enabling and prioritisation of resources), child food preferences (food choice), environmental norms (availability) and peer/social norms (food choice). The extensive number of key factors also supports the concept of whole school approaches to food, where a number of initiatives are most likely to have the greatest impact at disrupting the system; in this case, to positively influence child food choice across a school day. The analysis of the network should not be viewed in isolation. The different analytical methods and network statistics should be combined with the qualitative analysis and views of domain experts, to form a more holistic view of what factors are important. One potentially interesting finding from the core-periphery analysis is that the factors in the system that can be more easily influenced (such as ‘available funds resources’, ‘local authority buy in’, and ‘training provision and pay’) have grouped together into the middle layer (coreness ‘1’ in the table). This suggests that targeting a number of these factors together might provide an effective way of influencing the highly connected core of the network.

There has been limited stakeholder engagement work mapping school food systems in the UK, though previous work with School Food Trust staff working in this area has identified key factors that contribute to whether children eat a healthy lunch at school [31]. Our work builds upon this, by extending the focus of interest beyond healthy food provision to identification of key areas with a whole school approach to food. To the best of our knowledge, this is the first primary school food systems map for children aged 4 to 11 years that has been developed alongside key stakeholders in this area. Our thematic and network analysis findings also support (and extend) the systems work conducted within the Canadian school food system in which leadership, school food policies and priorities of stakeholders were deemed key factors [18]. Our CONNECTS-Food systems map builds on this by considering wider external factors that influence children’s food choice in schools; notably the influence of family circumstances, which has the potential to directly and indirect influence via multiple pathways (e.g. social norms, school meal uptake and free school eligibility).

Although there have been calls for the adoption of whole school approaches to food from the WHO [5], from national governments [6, 7, 13, 32] and other organisations [20, 33] here is limited evidence that this is embedded within the majority of schools. Given the potential extent of interventions associated with delivering whole school approaches to food, a lack of implementation is likely due to the associated perceived burden and cost against a background of lack of macro-level support and policy enforcement [18, 34]. However, research in this area suggests that, when well implemented, whole school approaches can have a substantial impact on diet, health [35] and food insecurity [36]. One example comes from the Daire randomised controlled trial, in which implementation of a multi-component school food intervention, ‘Nourish’, led to significant improvement in child emotional and physical health compared to non-intervention control schools [37]. Activities were delivered over a 2.5—5 month period and included improvements to the food environment, increased exposure to locally produced foods, sensory education and support for school food policy implementation. This demonstrates encouraging support for whole school approaches to food, though longer-term implementation and follow-up are needed to confirm sustainable impact. Other research evaluating a UK national systems based initiative called ‘Food for life’ indicates that children based at schools that have adopted the scheme eat more fruit and vegetables and are more likely to have a school meal [38], and systematic review evidences shows that implementation of school food environment policies can have a positive impact on diet quality in children [39, 40].

Our CONNECTS-Food systems map highlights some of the same factors previously hypothesised to influence the adherence to English School Food Standards [41] (which may be considered as a proxy to diet quality). These included factors within the physical environment (e.g. full production kitchens), support from head teachers, training for catering staff, low school prices and connections with the local authorities [41–43]. Other research has indicated that primary schools that adopt a whole school approach are more successful in adhering to the school food standards [9]. Ongoing research is being conducted in this area, including the FUEL study, which includes research to capture variation in the implementation of how the School Food Standards and the degree to which both of these has an impact on pupils’ dietary intake and dental health [44].

There are many strengths to this work. Perhaps most notable, we worked with a wide and extensive range of stakeholders from a variety of regions of the UK in the design and development of the system’s map including primary school children. Our group model building approach allowed all stakeholders to provide input and the online process further supported this by allowing a wider range of people to feed into the work remotely. However, given that stakeholders volunteered their time to take part in the workshops, it is recognised that they may have a bias view that does not necessarily represent the views of others. Further, as we were not able to fully clarify the strength of the relationships for all factors in our map, there are opportunities for future research to add greater depth in understanding of the school food system.

Interspersing our findings from the child in-person workshops within our adult stakeholder sessions (via a journey mapping process) also ensured that the child's voice was prominent throughout. However, whilst our online approach facilitated a wider range of stakeholder views, with good consideration of the relationship between factors, it was challenging for stakeholders to conceptualise the ‘strength of relationships’ in the given time. Had workshops been in-person, sufficient time would have been provided to facilitate this; however, we aimed to reduce online meeting fatigue via relatively short workshop session timings. Lastly, it is worth noting that our work was focused on primary schools. Although there are gaps in the evidence with regards to school food systems all schools (e.g. secondary/high schools) the approach to understand these would likely differ and would result in a different map, given how different these settings are. This remains to be a gap that we recommend is explore in future research.

## Conclusions

The CONNECTS-Food system map extends the current understanding of complexity within school food and key factors that influence children’s diets by highlighting how these relate to one another. This also enables us to explore the multiple social determinants of children’s diets, including family circumstances, social networks, peer influence, economic inequality and social capital. Importantly, doing this via a systems lens provides an opportunity to develop interventions that may have a positive impact on school food systems. Only focussing on one area, such as quality of school meals, is less likely to provide sustainable impact unless the whole school day and all stakeholders are focussed on the same goals. Achieving this can not be underplayed given the lack of resources and increased competing pressures within schools; however lessons can be learned from those who have managed to overcome barriers to successfully implement system wide, whole school approaches to food [15, 45–47]. Given the urgency of addressing the food system more widely, there is value in implementing these approaches so that both population and environmental health are considered [48, 49].

## Supplementary Information

**Additional file 1.** Workshop participants.

**Additional file 2.** CONNECTS food systems map: Domain, node and theme summaries.

**Additional file 3.** Sampling frame for recruitment.

## Data Availability

The datasets used and/or analysed during the current study are available from the corresponding author on reasonable request.
