# Supplementary material for: Understanding school food systems to support the development and implementation of food based policies and interventions
Source: Int J Behav Nutr Phys Act. 2023 Mar 13;20:29. doi: 10.1186/s12966-023-01432-2 (PMC10009978; doi:10.1186/s12966-023-01432-2)
Supplement: Supplementary file 1 — Additional file 1. Workshop participants. [file 12966_2023_1432_MOESM1_ESM.docx]

**Additional file 2. Workshop participants**

| Group concept mapping workshops - participant type | N = 81 |
| --- | --- |
| Teaching staff | N = 23 (Age range 25-64; 22 female, 1 male; 18 white British, 4 Asian/British Asian, 1 undeclared ethnicity) |
| Catering/lunch staff | N = 17 (Age range 35-64; 17 female; all white British/Irish) |
| Parent | N = 11 (Age range 35-64; 9 female, 2 male; all white British/Irish) |
| School governor | N = 7 (Age range 35-65+; 7 female; all white British) |
| Headteacher | N = 3 (Age range 50-64; 2 female, 1 male; all white British) |
| Food producer/distributor | N = 1 (Age range 35-49; female, white British/Irish) |
| Other (including representatives from local authorities, civil servants, school food organisations, nutritionists/dieticians) | N = 19 (Age range 18-65+; 14 female, 4 male; all white British/Irish) |
