# Supplementary material for: Understanding school food systems to support the development and implementation of food based policies and interventions
Source: Int J Behav Nutr Phys Act. 2023 Mar 13;20:29. doi: 10.1186/s12966-023-01432-2 (PMC10009978; doi:10.1186/s12966-023-01432-2)
Supplement: Supplementary file 2 — Additional file 2. CONNECTS food systems map: Domain, node and theme summaries. [file 12966_2023_1432_MOESM2_ESM.docx]

**Additional file 3**

**CONNECTS food systems map: Domain, node and theme summaries**

| **Domain** | **Node/Theme** | **Node/Theme summary** |
| --- | --- | --- |
| Leadership, culture and curriculum | Available funds/resources | Factors within this node pertained to potential barriers to implementing changes within schools to support a whole school approach to food (e.g., making changes to the dining area or school curriculum). This included lack of money, lack of time, lack of physical space or poor catering facilities. |
|  | Local authority buy-in | Stakeholders described potential variances between local authorities in their engagement with national school food initiatives (e.g. food for life award) and expectations around the implementations of whole school approaches to food (including extent to which this approach is expected and monitored). The importance placed on school food quality by the local authority was also felt to influence decision making around school catering contracts. Potential differences between local authority schools and academies were also discussed. Local authority buy-in was perceived to be somewhat dependent on local and national health priorities. |
|  | Extent of DfE/Ofsted monitoring | Stakeholders were in agreement that if implementation of school food policy, including a whole school approach to food, was monitored by Ofsted / DfE (i.e. affecting a school’s Ofsted rating), then head teachers and senior leaders would be more compliant / motivated to implement change. It was noted that expectations around whole school approaches to food currently differed between England, Scotland and Northern Ireland. |
|  | Priority of headteachers and senior leaders | Headteachers were described as being the “driving force” of the school. Headteacher priorities were perceived to be reflected throughout the school. For example, according to stakeholders, if headteachers placed importance on school meal uptake, healthy eating or pastoral care around food, then action to improve the school food system would be prioritised. Senior leadership were also described as being important for working with the headteacher to deliver their vision.  The headteacher was described as having autonomy to engage with school initiatives, including national school food initiatives, and that they could have the power to obtain / advocate for extra funding if needed in order to implement change. However, it was noted that they would first need to be aware of such initiatives and have a good understanding of their principles. Stakeholders felt headteacher awareness and understanding of initiatives (including a whole school approach to food) varied. |
|  | Priorities of school governors | The role of the school governor was described as being influential over headteacher and senior leadership priorities, as school’s need to “answer to” their governing body. |
|  | School food policy and culture | Stakeholders described a difference between schools in their implementation of school food policy and culture. For example, a school with a positive food culture may implement policies such as packed lunch policy, non-food rewards, water only, healthy celebration food etc. Some schools may also choose to engage in national initiatives (e.g. food for life) or provide the opportunity for children to take part in a garden club, allotment, extra curricular cooking lessons etc. It was noted that some initiatives and policies may be hard to sustain unless embedded within a positive food culture and supported by staff, pupils and parents. |
|  | Priorities and skills of teachers | Teachers were perceived to have autonomy over whether they incorporated food into their teaching. Teachers were also described as having autonomy to instigate initiatives, clubs and other activities to support a whole school approach to food. However, it was noted that some teachers may require training to increase either confidence around food incorporation into the curriculum, and that they need to be aware of healthy food initiatives in order to implement them. |
|  | Extend of food incorporation in curriculum | It was acknowledged that food is currently incorporated into the school curriculum to some extent in all schools, e.g. design and technology and science. But some schools / teachers choose to go beyond this to support a whole school approach to food. For example, allowing children to learn with and about food across the whole curriculum. Some stakeholders felt it was not clear exactly what children should be learning about food (e.g. nutrition, cooking skills, where food comes from, food science etc.), and that clearer guidance would be useful. It was also acknowledged that the school curriculum was already overloaded, making it difficult to add anything else. |
|  | Awareness of initiatives and resources | Stakeholders felt that there was a lack of awareness and knowledge around school food initiatives amongst some head teachers / teachers and other school stakeholders. There was also felt to be a large number of resources available to schools, potentially making it difficult to know which ones are most current, and / or of high quality. |
| Child food choices | Child food preferences and intake | Children were described as having the most control over what they eat during the school day: “they will not eat what they don’t like” (parent). Fussy eating was described as a common occurrence amongst children.  Food preferences and intake were described as being complex and influenced by many factors such as; exposure to healthy food at home and school, child mood, level of choice offered, education around what is healthy and sustainable, presence of developmental or sensory disorder, culture and body image. |
|  | Child hunger cues | Child hunger levels were named as influencing food preference and intake. Hunger cues were perceived as being influenced by many factors, including: activity levels, sleeping patterns, boredom, cues within the dining environment, weather, timing of meals and the amount of food eaten at home. |
|  | Peer/social norms | Children were described as being heavily influenced by their peers, particularly around school lunch time. Peer norms were described as influencing decisions around whether pack lunch or school lunch was chosen to be consumed, and the selection of packed lunch items.  Social norms were also described as influencing food choice. These included: gender norms; modelling from teachers, headteachers and parents; conditioning to view some foods as a treat/reward; and whether there was a culture of sitting down to eat meals together. Visiting a sweet shop or takeaway within the school day was also described as a social event. |
|  | Environmental prompts | Environmental prompts were described as influencing child food choice, including: the presence of shops, takeaways and ice cream vans within the locality of school and home; food marketing; celebrity influence; and access to supermarkets and grocers etc. |
| Home environment | Parental attitudes to school food policy | Parents were perceived by other stakeholders to sometimes “push-back” on school food policies, specifically around packed lunches. This, in turn, influenced implementation and sustainability of such policies. Parents themselves described how they were often confused by packed lunch policies, and felt that messages put out around healthy food from schools were often conflicting (e.g. banning chocolate in packed lunches, but then offering sweet desserts for school lunch). |
|  | Mode of travel to school | A child’s mode of travel to school was described as influencing a child's exposure to shops and takeaways on their journey to and from school. Mode of travel was also described as determining energy expenditure levels, which could influence hunger cues and food choice. |
|  | Urban vs rural location | Stakeholders discussed variances between living in an urban or rural location, including access and exposure to fast food outlets, supermarkets and leisure activities. Some stakeholders perceived households within a rural setting as needing to cook from scratch more, due to a lack of convenience shops and takeaways. More urban areas were perceived by some as having higher levels of deprivation. Food culture within urban locations was felt to differ from rural locations due to greater ethnic diversity. |
|  | Parent perception of school food quality and value | Stakeholders described how parental perceptions of school food quality influenced uptake of the school food offer, for example, if they felt that portion sizes were suitable, perceptions of the eating environment, and perceived quality of school meals. |
|  | Family circumstances and eating behaviours | Stakeholders described how eating habits at home were often reflected in eating habits at the school, particularly uptake of fruit and vegetables. Family circumstances in relation to income, health status and working patterns were thought to be influential over eating behaviours at home. |
| School food offer | Uptake of breakfast/after school club | Uptake of breakfast / after school club was thought to be influential over child food intake across the day. Typically the foods on offer during these times were described as starchy and of poor nutrient quality. Uptake of breakfast and after school clubs was described as being influenced predominantly by parental working patterns, but it was noted that some schools do offer a free breakfast to children. |
|  | School dining experience | The school dining experience was described by stakeholders as being particularly influential over whether children opted for school or packed lunch, and contributed to the overall food culture. Factors influencing the dining experience included long queues, influence of COVID-19, noise and time pressures. |
|  | Eligibility of free school meals | Eligibility and uptake of free school meals was described by stakeholders as influencing what children eat across the school day. Discussions were held amongst stakeholders around potential stigma of free school meals which potentially influenced uptake. |
|  | School/packed lunch uptake | Many factors were described by stakeholders as influencing school / packed lunch uptake, which, in turn, influenced child food intake accross the school day. These included, free school meal eligibility, convenience / time constraints, child food preference, and parental perceptions around what their children are most likely to eat. |
|  | School lunch menu  (Separated from quality of food provision as the menu options themselves highly influential offer uptake independently of quality) | The school lunch menu was felt to be an important component of the school food offer, which influenced child food intake across the school day. Stakeholders felt that menu development was influenced by the skills of the school cooks / catering companies. The meal options offered to children were described as being highly influential over whether children opted for school lunch. Some meals were described as always being more popular than others (e.g., pizza and roast dinner) |
|  | Quality of school food provision | Stakeholders felt that the quality of school food on offer was influential over school meal uptake. The quality of school food was described as being influenced by the cost of ingredients, skills of the school cooks, school food contracts and compliance with school food standards. It was noted that some schools were unable to prepare fresh food on site and therefore had to rely on food being brought in that was prepared off-site. |
|  | Skills/passion of cook and lunch staff | The skills and passion of the cook and lunch staff was described as contributing to the school food offer. Stakeholders also described that the school cook and lunch staff also had a larger role within the overall school food system. For example, school cooks and lunch time staff were described as influencing whether children tried new foods, the overall dining experience along with playing a role in the wider food culture. |
|  | Offer provided by catering companies | Stakeholders described that many schools rely on external catering companies to deliver their school food offer. This highlighted potential implications around the variability of quality and price. |
|  | Training provision and pay | Some stakeholders felt school catering teams were underpaid and undervalued, therefore highly skilled cooks may be put off from working in school due to low pay or unsuitable hours. It was also felt that highly skilled cooks often get promoted to managerial roles, removing them from the day to day running of the school kitchen. |
