# Supplementary material for: Understanding school food systems to support the development and implementation of food based policies and interventions
Source: Int J Behav Nutr Phys Act. 2023 Mar 13;20:29. doi: 10.1186/s12966-023-01432-2 (PMC10009978; doi:10.1186/s12966-023-01432-2)
Supplement: Supplementary file 3 — Additional file 3. Sampling frame for recruitment. [file 12966_2023_1432_MOESM3_ESM.docx]

Additional file 4. Sampling frame for recruitment

|  | IMD <8 | IMD ≥8 |
| --- | --- | --- |
| Urban location | 1 | 1 |
| Rural location | 1 | 1 |
| Clear food strategy | 1 | 1 |
| Food strategy not clear | 1 | 1 |
